# How Do Nutritionists/Dietitians Use Social Media to Communicate with Their Public? Global Perspectives on Social Media Practices: A Systematic Review

**DOI:** 10.3390/nu17223513

**Published:** 2025-11-10

**Authors:** Maria Gamito, Diana Rico Pereira, Mayumi Delgado, Filipa Vicente, Maria Leonor Silva, Paula Pereira

**Affiliations:** 1 Egas Moniz School of Health & Science, 2829-511 Caparica, Almada, Portugal; mgamito.nutri@gmail.com; 2 Nutrition Lab, Applied Nutrition Research Group, Egas Moniz Center for Interdisciplinary Research (CiiEM), Egas Moniz School of Health & Science, 2829-511 Caparica, Almada, Portugal; mayumidelgado@gmail.com (M.D.); fvicente@egasmoniz.edu.pt (F.V.); lsilva@egasmoniz.edu.pt (M.L.S.); pmpereira@egasmoniz.edu.pt (P.P.)

**Keywords:** social media, nutritionist, dietitian, social media use practices, digital nutrition communication, systematic review

## Abstract

**Background:** Social media has emerged as a powerful communication tool for healthcare professionals, including nutritionists and dietitians, particularly since the COVID-19 pandemic. Evidence suggests that their online presence can enhance nutritional literacy and play a crucial role in countering misinformation. **Objective:** This systematic review aims to investigate how and why Registered Nutritionists and Dietitians (RNDs) use social media in their professional practice, focusing on benefits, challenges, and impact. **Methods:** A systematic literature search was conducted between 1 January 2019 and 28 February 2024, in PubMed, Scopus, Scholar, and SciELO databases using terms such as ‘nutritionist’, ‘dietitian’, and ‘social media’. Quality was assessed using the MMAT tool. This review followed the Preferred Reporting Items for Systematic Reviews and Meta-Analysis (PRISMA) guidelines. The included studies were analysed with respect to their content, professional practices, and patterns of social media use. **Results:** Of the 359 articles identified through the systematic search, 10 cross-sectional studies conducted using questionnaires were included in this review. Sample sizes ranged from 10 to 2542 participants across nine countries. Instagram and Twitter were the most frequently used platforms among RDNs, primarily for sharing evidence-based nutritional information, counselling content, and professional promotion. Reported usage ranged from 37.5% to 100%, with a marked increase during the COVID-19 pandemic, especially among younger professionals. Key enablers included enhanced communication, professional visibility, and cost-effective outreach, while main challenges involved limited digital literacy and difficulties replicating face-to-face counselling online. Although ethical concerns were reported, many RNDs maintained compliance with professional standards, particularly in regions with strict marketing regulations. **Conclusions:** This systematic review provides evidence that social media is a valuable tool for RNDs, particularly in the context of food and/or nutritional education. RNDs would benefit from training in content creation, knowledge dissemination and ethical digital communication. However, clearer guidelines from professional organisations are also recommended.

## 1. Introduction

Social media currently has a strong global presence, offering healthcare professionals an opportunity to engage with the public [1]. Its use can enhance healthcare by supporting professional development, facilitating doctor–patient communication, and advancing health research [2]. Furthermore, social media also serves as a platform for the exchange of health information, with the potential to improve health outcomes. However, the study by Moorhead et al. reported that safeguarding the high quality, credibility, and confidentiality of shared information is imperative for maintaining public trust and supporting evidence-based public health communication [3].

In this context, the active presence of qualified professionals in nutrition sciences across online platforms has proven to be an effective strategy to increase food literacy and promote evidence-based knowledge on healthy eating. Moreover, Diekman et al. verified that their presence helps counteract misinformation frequently disseminated by individuals lacking credentials in nutrition and dietetics [4]. Therefore, to ensure the credibility of shared content, the active presence of qualified nutrition professionals is essential, specifically Registered Nutritionists and Dietitians (RNDs).

According to Sbardelotto et al. [5] and Bookari et al. [6], the COVID-19 pandemic marked a significant shift in the online presence and remote working practices of RNDs. During and following the pandemic, the frequency of nutrition-related content shared by RNDs on social media increased substantially [5,6]. Several studies have examined the effectiveness of social use and communication strategies employed by RNDs, including efforts to develop appropriate evaluation tools. However, these models still require refinement [7,8,9,10].

RNDs have utilised social media to promote their services [11], connect and engage with peers, discuss nutrition-related topics [5], and provide client support via online platforms [11]. However, many of these studies focus on narrowly defined populations, such as RNDs from a specific country [5,12,13,14] or a particular area of expertise [11], which limits their generalizability to the broader international RND community [13,14]. In Probost et al. study, the results suggest that ongoing monitoring of social media use among RNDs is essential to detect changes in communication patterns and influence networks [7]. Few reviews have explored global trends, ethical considerations, and the impact of RND social media use across regions. Therefore, this systematic review synthesises evidence on how RNDs use social media professionally across diverse settings.

## 2. Materials and Methods

### 2.1. Search Criteria and Strategy

This systematic review followed the Preferred Reporting Items for Systematic Reviews and Meta-Analysis (PRISMA) guidelines and was registered with the International Prospective Register of Systematic Review (https://www.crd.york.ac.uk/PROSPERO/; accessed on 22 July 2025; registration no. CRD420251027216). MG and DP conducted the methodological process in accordance with the PRISMA guidelines, while MLS and PP ensured compliance with these standards. The research question and specific objectives were defined before the search process. The research question was addressed by examining the following variables: professional profiles, most commonly used social media platforms, types of content shared, and attitudes and perceptions regarding social media use.

The literature search was conducted from 1 January 2019 to 28 February 2024, across the following databases: PubMed, Scopus, Google Scholar, and SciELO. The search strategy was applied to titles and abstracts using predefined keywords: (“nutritionist” OR “dietitian” OR “dietician”) AND (“social media” OR “digital media” OR “Facebook” OR “Instagram” OR “Twitter” OR “WhatsApp” OR “social network” OR “online network”). No filters were selected for the search.

The time frame was selected to reflect the post-COVID-19 period. All retrieved articles were imported into Mendeley, and duplicates were removed using the software’s built-in function. Additionally, a manual search of the references listed in the included studies was conducted to identify any additional relevant publications.

### 2.2. Studies Selection

Two independent researchers (MLS and PP) conducted the initial literature search and screening based on titles and abstracts. Full-text articles were subsequently assessed for eligibility according to predefined inclusion and exclusion criteria. In cases of disagreement, all researchers reviewed the article and reached a consensus.

The Population, Exposure, Comparator, and Outcomes (PECO) framework was used to structure the data extraction, despite the absence of a comparator:

P (Population): Registered Nutritionists and Dietitians (RNDs)

E (Exposure): Use of social media in professional practice

C (Comparator): Not applicable

O (Outcome): Promotion of services, enhancement of nutritional literacy, countering misinformation, adherence to best practices, and professional challenges (e.g., lack of training, ethical concerns).

**Figure 1:**
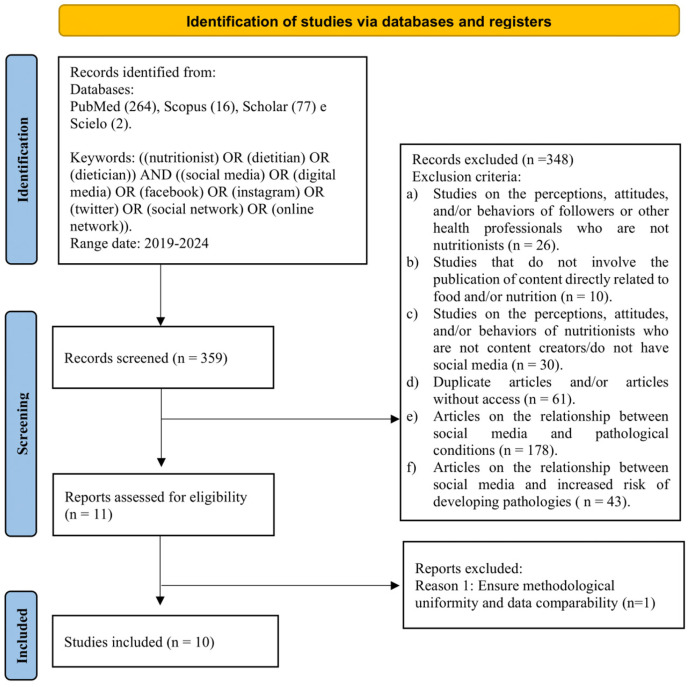
PRISMA flowchart of the study selection process.

Relevant data from included studies were extracted and included in the review. The screening and selection process is illustrated in the PRISMA flowchart (Figure 1).

### 2.3. Eligibility Criteria

Inclusion criteria:

- Peer-reviewed studies published in English.
- Studies involving RNDs who act as content creators in any field of nutrition (e.g., sports nutrition, pediatric nutrition).
- Studies focused on the use of social media platforms.
- Studies with quantitative, qualitative or mixed methods designs.

Exclusion criteria:

- Studies that did not involve the publication of content directly related to food and/or nutrition.
- Studies in which content creators did not use social media.
- Studies focusing on the relationship between social media and pathological conditions or increased risk of developing pathologies.

### 2.4. Quality Assessment

The methodological quality of the studies included in this review was assessed using the Mixed Methods Appraisal Tool (MMAT, version 2018) [15], applying the specific evaluation criteria relevant to each study design. The initial appraisal was performed by DRP and independently verified by two additional reviewers (MLS and PP). MD resolved disagreements. Based on the MMAT quality classification, the methodological standards of the selected studies ranged from poor to good. MMAT scoring was defined as poor (0–50%), fair (51–75%), and good (76–100%), according to Almutairi et al. [16].

### 2.5. Data Extraction and Synthesis

Data extraction was independently conducted using Microsoft Excel 365 (Microsoft Corporation, Washington, DC, USA). Qualitative data were analysed thematically, and quantitative findings were summarised descriptively. The analysis focused on identifying general characteristics of the selected studies and mapping the following outcomes and measures, respectively:

The table in Section 3.3: Country, study design, sample size and study aim.

The table in Section 3.4: Participant characteristics (age and gender) (measured as percentage and mean).

The table in Section 3.5: Use of social media by RNDs in professional practice across various platforms (measured as percentage of usage).

The table in Section 3.6: Nutrition content and information shared via social media (measured as percentage).

The table in Section 3.7: Motivators, challenges, barriers, and opinions related to social media use.

A quality content analysis was also conducted to identify common practices and patterns in the professional use of social media by RNDs.

## 3. Results

### 3.1. Literature Search

A total of 359 articles were identified across the four databases: PubMed (264), Scopus (16), Scholar (77), and SciELO (2). After removing duplicates and screening the titles and abstracts, 11 full-text articles were selected for further assessment of eligibility. Following the application of inclusion and exclusion criteria, 10 articles were included in the final review. Of the 359 records identified, 349 studies were excluded after screening. The selection process is illustrated in the PRISMA flow diagram (Figure 1).

### 3.2. Quality Scores of Studies

Seven studies were assessed according to the criteria for quantitative descriptive designs, while three were evaluated using the mixed methods framework, in line with MMAT 2018 classification. Among the ten included studies, one received a score of 40%, three were rated between 60% and 75%, and six achieved the maximum score of 100%. A detailed summary of the appraisal results is presented in Table 1.

### 3.3. Characteristics of the Included Studies

As presented in Table 2, the observational studies included in this systematic review (*n* = 10) predominantly used questionnaires for data collection, with most administered online. The studies were published from 2019 to 2024. Two notable exceptions were identified: the study by Zielińska-Tomczak et al. (2021) [8], which analysed Instagram profiles of RNDs, and the conference paper by Saboia et al. (2021) [10], which combined a questionnaire with a literature review.

Geographically, the studies were conducted across several continents, reflecting a diverse international scope: Europe (*n* = 4), Africa (*n* = 1), Oceania (*n* = 2), America (*n* = 1), and Asia (*n* = 2).

### 3.4. Profiles of the RNDs Participants by Gender and Age

The total sample sizes of the included studies ranged from 30 to 2542 participants (Table 1). In terms of gender, a consistently high proportion of female participants was reported, ranging from 80% to 97.9% (Table 2). These findings align with the broader demographic profile of the RND profession, which is predominantly female.

Participants’ ages ranged from 26 to over 65 years, with most studies reporting a mean age between 27 and 40.5 years. Notably, not all participants were young adults; for example, 32.6% of respondents were between 35 and 64 years of age and reported active engagement in professional practice (Table 3).

Some studies did not report participant age [8,9,11], and in the studies by Dunne et al. (2019) and Squires et al. (2023), gender was also not specified, which limits the depth of comparison across all studies [9,11]. Nevertheless, among the studies providing demographic details, a clear trend emerged: participants were predominantly younger and female. This trend may influence both the type of content shared on social media and the communication strategies adopted. Younger professionals are likely to be more familiar with digital tools and social media platforms, which may facilitate greater adaptability and more effective use of these channels for professional purposes.

### 3.5. Analysis of Social Media Practices and Use

Table 4 presents the proportion of RND participants in each study who reported using social media in their professional practice, along with the percentage distribution across various digital platforms. In most studies, a high percentage of RNDs reported using social media for professional purposes. However, a study by Probst and Peng (2019) had shown a lower rate of professional social media use (37.5%) [7]. Instagram emerged as the most frequently studied platform (*n* = 9), with three studies specifically focusing on its use by RNDs.

The results reporting 100% of RNDs using social media in their professional practice were those that exclusively targeted existing RND profiles on Instagram— for example, 10 Instagram profiles [8], 163 RNDs active on Instagram [12], and 50 Instagram profiles [9]. However, when comparing the use of different social media platforms, no significantly consistent patterns were observed across most studies. The study by Bookari et al. (2023) [6] primarily aimed to compare social media use between the period before the COVID-19 pandemic and during the pandemic. The results indicated that usage during the pandemic was higher (80%) compared to the pre-pandemic period (68.9%) [6]. These findings contribute to an understanding of the impact of the COVID-19 pandemic on increasing communication and social media engagement among RNDs. This phenomenon is likely explained by the quarantine measures, which prompted a shift towards online communication.

In the remaining studies, the proportion of RNDs using social media in their professional practice ranged from 37.5% [7] to 79.2% [14]. Due to variability in sample sizes and study designs, it is not possible to establish a clear trend in the increase or decrease in professional media use. However, except for the study by Probst and Peng (2019), the majority of studies reported that over half of participants used social media in their professional practice [7]. This observation supports the growing relevance of online platforms in contemporary nutrition and dietitians’ practice.

The studies by Dunne et al. (2019) [11] and Visser et al. (2024) [14] present results across all the social media platforms identified in Table 3, enabling comparisons between them. In the study by Dunne et al., Twitter was the most frequently used platform among RNDs, with 100% of participants reporting its use for professional purposes [11]. Conversely, in the study by Visser et al. (2024) [14], Instagram was the preferred platform among participants, with a usage rate of 45.5% [11]. These differences may be explained by geographical variation in platform preferences; for example, Twitter appears to be more commonly used in England, whereas Instagram is more relevant among South African professionals. This distinction may also reflect a broader trend towards increased Instagram use and declining engagement with Twitter among RNDs.

Some studies suggest that the reluctance of certain RNDs to adopt social media may be linked to concerns regarding the effectiveness of its use. In one study, 58.8% of RNDs reported perceiving face-to-face counselling as more effective than online interactions [12]. Furthermore, in the same study, 70.2% of the respondents considered much of the content shared online to be irrelevant or unreliable. Such perceptions may help explain the limited engagement of some RNDs with social media platforms.

In summary, social media practices and uses ranged from 37.5% to 100%, with Instagram and Twitter being the most widely used platforms. This indicates a wide range of variability in how RNDs utilise social media.

### 3.6. Themes and Shared Content

Based on the selected studies, three main themes emerged as the most frequently shared by RNDs across different social media platforms (Table 5). The most commonly shared content was related to nutritional information or factual posts (*n* = 7). The second most frequent content concerned counselling, dietary planning, and/or promotion of professional activities (*n* = 6). Food-related content, including recipes and cooking methods, was also reported in several studies (*n* = 5).

The presentation of results varied across studies, and participant sample sizes differed considerably. In the study by Bookari et al. (2023), a comparison between pre- and post-pandemic contexts revealed that content related to counselling, planning, and/or promotion of professional activities was the most frequently shared (Pre-COVID—51.1%; Post-COVID—63%), while nutritional information/facts was the second most common theme (Pre-COVID—35.7%; Post-COVID—44.1%) [6]. In Dunne et al. (2019) [11], the themes shared by participants were analysed across various online platforms. Nutritional information or factual posts emerged as the most frequently shared content, particularly on Twitter (79%). Recipes were primarily shared via WhatsApp (59%), as were nutritional plans (44%) [11]. For instance, in Visser et al. (2024), nutrition information and factual content were the most commonly shared themes (62.2%), followed by counselling-related content (60.6%), which was more frequently shared on Facebook [14]. Similarly, Liikkanen et al. (2024) [13] found that 46% of Finnish RNDs used Twitter to share evidence-based nutritional information, while another 46% shared cooking-related content on Instagram. Notably, nutritional counselling appeared less frequently in this study, being shared primarily on YouTube (17%) and Instagram (4.5%) [13]. These findings contrast with the higher prevalence of counselling content reported in studies by Dunne et al. (2019) [11] and Visser et al. (2024) [14].

Overall, Twitter remains one of the preferred platforms for RNDs to share nutritional information or factual content, although this trend is not reflected in the other two themes. According to Dunne et al. (2019), WhatsApp was the most frequently used to share recipes (59%) and nutritional plans (44%) [11]. In contrast, Liikkanen et al. (2024) observed that Instagram was more commonly used to share cooking-related content (46%), while YouTube had the highest proportion of counselling-related content (17%) [13].

The findings of this systematic review support the hypothesis that nutritional information or factual content is the predominant theme shared by RNDs on social media as part of their professional practice. Studies such as those by Dunne et al. (2019) and Liikkanen et al. (2024) suggest that Twitter is the preferred platform for disseminating this type of content [11,13]. Likewise, Instagram appears to be increasingly favoured among RNDs for professional content sharing, which suggests that RNDs are leveraging visual content to enhance their communication strategies and engage with their audience more effectively. RNDs primarily focus on sharing reliable nutritional information, which highlights their role in promoting health and wellness through social media.

### 3.7. Attitudes and Opinions of RNDs Regarding Social Media

Among the ten studies included in this review, six presented relevant findings on the attitudes and opinions of RNDs towards the use of social media [5,6,10,11,13,14]. The most frequently assessed aspects were related to the RNDs’ digital competencies, the perceived importance of social media in their professional careers, and their awareness of and compliance with relevant guidelines. Additionally, RNDs identified both facilitators and barriers to the use of these platforms, which are summarised in Table 6, under three main categories: (1) motivating factors and enablers, (2) challenges and barriers, and (3) perceived importance/opinions/benefits.

Several studies, such as Dunne et al. (2019) [11], organised findings into two broad dimensions: enablers and challenges. Enablers helped to explain how and why RNDs use social media in professional contexts, while challenges reflected limitations, ethical concerns, and practical difficulties associated with digital engagement [11]. Saboia et al. (2021) [10] developed an online survey using a Design Thinking approach to investigate the use of social media by Portuguese RDNs. The study explored user behaviour, the perceived relevance of these platforms for disseminating scientific knowledge and promoting their profession, as well as ethical considerations in online interactions [10]. Across the studies, a range of enabling factors emerged. These include improvements in communication and visual learning [11], as well as the integration of social media into daily professional routines [13]. Social media was also viewed as a cost-effective tool, offering broad reach without the limitations of geographic distance or scheduling constraints [6].

However, several challenges and barriers were consistently reported across studies. The most frequently cited was a lack of technical knowledge and inexperience in managing social media accounts [6,11,14]. Other concerns included communication difficulties resulting from the absence of face-to-face interactions and the inability to conduct anthropometric assessments in a virtual context [6]. Ethical concerns, emotional impacts, and the risk of unfavourable professional comparisons were also noted [5]. Despite these challenges, many RDNs recognised the value and importance of social media for professional practice. It was perceived as an effective tool for promoting services [5,6,10,11,13,14], disseminating scientific information, and engaging with both the public and the professional peers [10]. These platforms were also seen as beneficial for expanding outreach and enhancing professional visibility [6].

Collectively, the studies by Dunne et al. (2019), Saboia et al. (2021), Sbardelotto et al. (2022), Liikkanen et al. (2024), Visser et al. (2024) and Bookari et al. (2023) provided a nuanced understanding of RDNs’ attitudes towards social media [5,6,10,11,13,14]. Employing diverse methodologies—including online questionnaires, observational analyses, and participatory design—these studies highlight the perceived professional benefits of social media use, such as client engagement and the dissemination of evidence-based information.

Simultaneously, they highlight the need for ongoing support and training, particularly in areas such as ethical practices, digital literacy, and misinformation management, to ensure the responsible and effective integration of social media into dietetic practice.

Overall, the RNDs reported that social media is a valuable tool for a professional approach. Specifically, RNDs have identified benefits, including sharing evidence-based content, enhancing client engagement, and facilitating professional networking.

#### 3.7.1. Social Media Skills

The use of social media has emerged as a significant factor in professional practice among RNDs. However, a lack of training in content creation and public communication remains one of the main barriers to its practical use. This issue was highlighted in the study by Dunne et al. (2019) [11], in which only 5% of participants reported having received formal training in social media and online interventions. Nevertheless, 84% expressed interest in receiving such training [11]. Regarding digital abilities, the results suggest that younger professionals tend to demonstrate greater proficiency in using social media compared to those over 45 years old [13]. This may be attributed to their early exposure to and familiarity with technology and digital platforms. Therefore, offering targeted training opportunities, particularly for RNDs aged 45 and above, could be a significant step towards promoting digital engagement and competence within the profession.

#### 3.7.2. Importance of Social Media for RND’s Careers

The importance of social media in the professional careers of RNDs was also evaluated. Evidence suggests that RNDs are increasingly aware of the need to maintain a professional online presence, recognising its role in communication, education and the promotion of services [5,10]. According to the study by Sbardelotto et al. (2022) [5], 78.5% of the 227 RND participants agreed that social media is an excellent tool for promoting their services (see Table 5). Additionally, 45% believed that using social media offers a commercial advantage over those who do not engage with these platforms, while 41.9% agreed that social media is essential for promoting health [5]. Similarly, in a study of Saboia et al. (2021) [10], RNDs expressed strong support for the role of social media in professional practice. Among the 16 participants, several ‘completely agreed’ that social media is essential for both disseminating information about healthy eating and sharing scientific knowledge related to nutrition [10].

#### 3.7.3. Ethical Practices for Social Media

Ethical obligations related to the use of social media were a key aspect evaluated to better understand RNDs’ attitudes, reinforcing the importance of adhering to professional ethical standards. In the study by Sbardelotto et al. (2022), 177 out of 289 participants (61.2%) believed that most professionals do not comply with the ethical guidelines established by the Regional Council of Dietitians (RCD) [5]. In contrast, findings from Visser et al. (2024) indicated that the majority of South African RNDs are aware of and comply with the ethical standards set by the Health Professions Council of South Africa (HPCSA) [14].

However, concerns persist regarding unethical practices on digital platforms, including inappropriate advertising and a lack of scientific references in shared content. The results underscore the importance of targeted training to ensure RNDs adhere to ethical and legal standards, particularly in relation to confidentiality, data privacy, and professional conduct. This includes avoiding sharing identifiable case studies without explicit consent. It is essential to safeguard personal data collected through social media interactions, ensuring that any sensitive information is securely handled and stored. RNDs should verify the accuracy of the information shared on social media to prevent the spread of misinformation. This includes providing evidence-based nutritional information and clearly referencing credible sources. The HPCSA guidelines were also noted for their relative strictness, especially regarding restrictions on specific marketing strategies.

Overall, these findings suggest that compliance with social media guidelines among RND’s remains inconsistent, underlining the need for further research and education to address this gap and promote responsible professional behaviour online.

## 4. Discussion

This systematic review examined the practices and uses of social media among Registered Nutritionists and Dietitians (RNDs), synthesising data from ten studies. The findings reveal a consistent use of platforms such as WhatsApp, Facebook, Twitter, LinkedIn and Instagram across a range of professional contexts, highlighting Instagram and Twitter as the most widely used. However, direct comparisons between studies should be interpreted with caution due to methodological heterogeneity and variation in sample characteristics.

Most of the included studies employed online questionnaires as the primary data collection method and were published between 2019 and 2024, reflecting a growing academic interest in this area. The studies span five continents—Europe (5), Africa (1), Oceania (2), America (1), and Asia (2), demonstrating a relatively balanced geographical distribution. All studies utilised a cross-sectional observational design, providing insights into current practices and perceptions among RNDs. Notably, Zielińska-Tomczak et al. (2021) [8] conducted a direct analysis of Instagram profiles, while Saboia et al. (2021) [10] combined questionnaires with a literature review, offering more comprehensive perspectives.

A prior scoping review by Dumas et al. (2018) highlighted the lack of research in this area and noted that the use of social media in dietetic practice was still in its early stages [17]. Another review conducted by Saboia et al. (2020) [1] included nine studies published between 2013 and 2018, primarily from high-income countries such as the USA, Canada, Ireland, and the UK, suggesting that the field initially developed in more resource-advantaged contexts. While earlier studies noted limited RNDs online engagement, post-2020 data show increased digital participation.

Sample sizes in the included studies ranged from 30 to 2542 participants, with a strong predominance of female professionals (80% to 97.9%), reflecting the gender profile of the profession. Participants’ ages ranged from 26 to 65 years, with a substantial proportion (32.6%) aged between 35 and 64 reporting active use of social media.

Reported usage rates ranged from 37.5% to 100%, demonstrating a widespread integration of social media into professional routines. Instagram emerged as the most frequently analysed platform, especially during the COVID-19 pandemic. Regional variation was also evident, with Twitter being more popular in England [11], while Instagram was more popular in South Africa [14].

Despite high usage rates, some RNDs expressed scepticism about the efficacy of social media, particularly the traditional face-to-face counselling. Concerns regarding the reliability of information were common, especially given the anonymity of online authorship, which complicates content validation [12]. Additionally, ethical concerns, such as the protection of client confidentiality and privacy, present further challenges in the digital environment [18].

This systematic review revealed that sharing nutritional information is the most common professional activity on social media (*n* = 7), followed by counselling, planning, and professional self-promotion (*n* = 6), while food-related content, such as recipes, is less common (*n* = 5). Twitter has traditionally been used for disseminating evidence-based information, although its use has declined (from 79% to 46%) [11,13]. A notable increase in content related to professional promotion was observed during the pandemic, reflecting a shift in how RNDs leveraged social media for visibility and engagement.

Importantly, different platforms appear to serve distinct professional purposes: Pinterest is primarily used for patient education, Twitter for sharing news and engaging in conferences, and LinkedIn for career development [2]. These findings reinforce the importance of adopting platform-specific strategies in professional digital communication.

A significant barrier identified was the lack of formal training in content creation and public communication. For instance, Dunne et al. (2019) found that only 5% of participants had received social media training, although 84% expressed interest in such programs [11]. Younger professionals demonstrated greater ease with digital tools, likely due to earlier exposure to technology. Therefore, targeted training, particularly for older RNDs, could help bridge the skills gap and promote more effective and responsible engagement.

Social media is widely recognised as an effective tool for promoting services, sharing scientific knowledge, and engaging with the public and peers [19]. Several studies highlight its commercial advantages and positive impact on professional visibility [4,5,10]. In order to interact with their audience on social media, health advocates should modify their messages to fit the various platforms and employ tactics linked to increased interaction [20].

Nevertheless, adherence to ethical guidelines remains inconsistent. While South African RNDs reportedly comply with the strict HPCSA regulations, concerns about inappropriate advertising and the absence of scientific referencing were prevalent in other regions, as noted by Sbardelotto et al. (2022) [5]. These inconsistencies underscore the need for comprehensive training on digital ethics and legal responsibilities, particularly in relation to confidentiality and data privacy [21].

The study’s limitations are related to the fact that all studies employed a cross-sectional methodology and the variation in sample sizes, which can affect the comparability and generalizability of the results. Moreover, the reliance on self-reported data gathered through questionnaires, especially those administered online, increases the risk of bias or erroneous reporting, which could compromise the reliability and validity of the results.

The disparity in the reporting of outcomes among the included studies should also be analysed carefully, specifically in terms of participant age, which was presented in different formats. Some studies reported mean values, while others provided percentages for specific age groups. This variation in reporting methods makes it difficult to coherently determine the age profile of RNDs using social media in their practice, thereby hindering meaningful comparative analysis.

Nevertheless, the limitations of this review contribute to clarifying the importance of a social media practices approach and allow future research. Further studies should adopt longitudinal designs to assess the long-term impacts of social media use among RNDs.

Although the benefits are evident, including greater professional visibility and more efficient dissemination of information [22,23], significant gaps remain in terms of training, ethical compliance, disinformation and platform-specific literacy [4,24,25]. Therefore, this systematic review encourages the ethical use of social media and platforms in the professional context of RNDs.

## 5. Conclusions

Overall, this review highlights the increasing significance of social media in dietetic practice and its diverse applications across various professional settings. Social media, especially Instagram and Twitter, is widely used for patient education, evidence-based communication, and professional branding. Most studies have found regular social media use among RNDs, with usage influenced by age, digital skills, and regional norms. Training in digital communication and content validation is necessary, along with guidance on confidentiality and the prevention of misinformation. Longitudinal studies are needed to evaluate client outcomes, identify differences in generational engagement, and investigate the development of digital identity.

## Figures and Tables

**Table 1:** Studies quality assessment scoring using MMAT [15].

	4. Quantitative Descriptive Studies					5. Mixed Methods Studies					Quality Score (%)
**References**	**4.1**	**4.2**	**4.3**	**4.4**	**4.5**	**5.1**	**5.2**	**5.3**	**5.4**	**5.5**	
Dunne et al. (2019) [11]						Yes	Yes	Yes	Yes	Yes	100% good
Probst e Peng (2019) [7]						Yes	Yes	Yes	Yes	Yes	100% good
Zielińska-Tomczak et al. (2021) [8]						Yes	Yes	Yes	Yes	Yes	100% good
Saboia et al. (2021) [10]	Yes	No (pilot study)	Yes	No	No						40% poor
Sbardelotto et al. (2022) [5]	Yes	Yes	Yes	Yes	Yes						100% good
Khadem Al-Hoseini et al. (2023) [12]	Yes	No	Yes	Can’t tell	Yes						60% fair
Squires et al. (2023) [9]	Yes	Yes	Yes	Yes	Yes						100% good
Bookari et al. (2023) [6]	Yes	Can’t tell	Yes	Yes	Yes						75% fair
Liikkanen et al. (2024) [13]	Yes	No	Yes	Can’t tell	Yes						60% fair
Visser et al. (2024) [14]	Yes	Yes	Yes	Yes	Yes						100% good

(4.1. Is the sampling strategy relevant to address the research question? 4.2. Is the sample representative of the target population? 4.3. Are the measurements appropriate? 4.4. Is the risk of nonresponse bias low? 4.5. Is the statistical analysis appropriate to answer the research question? 5.1. Is there an adequate rationale for using a mixed methods design to address the research question? 5.2. Are the different components of the study effectively integrated to answer the research question? 5.3. Are the outputs of the integration of qualitative and quantitative components adequately interpreted? 5.4. Are divergences and inconsistencies between quantitative and qualitative results adequately addressed? 5.5. Do the different components of the study adhere to the quality criteria of each tradition of the methods involved?).

**Table 2:** Design and characteristics of the studies included in the systematic review.

Authors	Country	Journal	Study Design	Sample Size	Aim
Dunne et al. (2019) [11]	UK	Journal of Sports Sciences	Cross-sectional study (Online questionnaire)	44	Explore how sports nutritionists use social media and gather opinions and experiences regarding its use in professional practice.
Probst & Peng (2019) [7]	Australia	Nutrition & Dietetics	Cross-sectional study (Online questionnaire)	340	Determine the influence level of dietitians on social media and assess a metric tool (NodeXL) to measure this influence.
Zielińska-Tomczak et al. (2021) [8]	Poland	Nutrients	Cross-sectional study (Analysis of Instagram profiles)	10 Instagram profiles	Examine the use of the Kirkpatrick Model versus the New World Kirkpatrick Model to evaluate the effectiveness of educational nutrition content shared on social media.
Saboia et al. (2021) [10]	Portugal	Procedia Computer Science (Conference paper)	Cross-sectional study (Literature review and questionnaire)	30	Describe the creation process of an online questionnaire for Portuguese dietitians/nutritionists using the Design Thinking method.
Sbardelotto et al. (2022) [5]	Brazil	JMIR Formative Research	Cross-sectional study (Online questionnaire)	288	Describe the role of social media in professional practice, dietitians’ perceptions and behavioural changes during the COVID-19 pandemic in Brazil.
Khadem Al-Hoseini et al. (2023) [12]	Iran	Research Square	Cross-sectional study (Online questionnaire)	131	Evaluate Iranian dietitians’ opinions on virtual nutrition counselling via Instagram.
Squires et al. (2023) [9]	Australia	Nutrition & Dietetics	Cross-sectional study (Checklist questionnaire)	50 (25 RNDs & 25 SDs)	Develop a tool to assess the ethical and professional practices of dietitians and dietetic students on social media.
Bookari et al. (2023) [6]	Saudi Arabia	Frontiers in Public Health	Cross-sectional study (Questionnaire)	2542	Understand nutritionists’ perspectives and practices regarding the use of social and mass media during the shift to tele-nutrition in the COVID-19 era.
Liikkanen et al. (2024) [13]	Finland	Journal of Human Nutrition and Dietetics	Cross-sectional study (Online questionnaire)	107	Describe how Finnish dietitians use different social media channels in their professional practice and assess their digital competencies.
Visser et al. (2024) [14]	South Africa	South African Journal of Clinical Nutrition	Cross-sectional study (Online questionnaire & platform review)	125	Investigate the use of social media and electronic communication by South African dietitians and their adherence to the HPCSA ethical guidelines.

**Table 3:** Description of participant characteristics according to gender and age, across the included studies.

Authors	Gender	Age
Dunne et al. (2019) [11]	Not mentioned	Not mentioned
Probst & Peng (2019) [7]	Female: 97% Male: 3%	26–35 years: 44.8% 36–45 years: 51%
Zielińska-Tomczak et al. (2021) [8]	Female: 90% Male: 10%	Not mentioned
Saboia et al. (2021) [10]	Female: 80% Male: 20%	Mean: 27 years
Sbardelotto et al. (2022) [5]	Female: 97.9% Male: 2.1%	Mean: 29 years
Khadem Al-Hoseini et al. (2023) [12]	Female: 87.8% Male: 12.2%	Mean: 30.8 years
Squires et al. (2023) [9]	Not mentioned	Not mentioned
Bookari et al. (2023) [6]	Female: 88.2% Male: 11.8%	18–34 years: 67.1% 35–64 years: 32.6% ≥65 years: 0.3%
Liikkanen et al. (2024) [13]	Not mentioned	Mean: 40.5 years
Visser et al. (2024) [14]	Female: 96.0% Male: 3.2%	Mean: 35.4 years

**Table 4:** Percentage of Registered Nutritionists and Dietitians (RNDs) reporting the use of social media/digital platforms in their professional practice.

Authors	Social Media (%)	Instagram (%)	Facebook (%)	Twitter (%)	LinkedIn (%)	WhatsApp (%)	Others (%)
Dunne et al. (2019) [11]	89%	68%	93%	100%	86%	86%	52%
Probst & Peng (2019) [7]	37.5%	NM	58%	Yes (not quantified)	NM	NM	NM
Zielińska-Tomczak et al. (2021) [8]	100% ^1^	100%	NA	NA	NA	NA	NA
Saboia et al. (2021) [10]	NM	97.0%	80%	NM	NM	77.0%	NM
Sbardelotto et al. (2022) [5]	91.7%	84.8%	1.5%	0%	0.8%	11%	1.9%
Khadem Al-Hoseini et al. (2023) [12]	100% ^1^	100%	NA	NA	NA	NA	NA
Squires et al. (2023) [9]	100% ^2^	100%	NA	NA	NA	NA	NA
Bookari et al. (2023) [6]	BC: 68.9% AC: 80%	BC: 47.1% AC: 51.7%	BC: 37.8% AC: 40.9%	BC: 18.5% AC: 21.1%	BC: 6% AC: 8.3%	BC: 2.7% AC: 2.2%	NM
Liikkanen et al. (2024) [13]	58%	71%	89.0%	35.0%	68%	NM	19%
Visser et al. (2024) [14]	79.2%	45.5%	31.6%	2.1%	0%	6.1%	14.3%

(NA = Not Applicable; NM = Not Mentioned; BC = Before COVID-19; AC = After COVID-19); ^1^ Studies exclusively included RDNs who actively used Instagram. ^2^ Study included only RDNs who used social media, without specifying the platform.

**Table 5:** Percentage of Registered Nutritionists and Dietitians (RNDs) by type of content shared on each social network.

Authors	Nutrition Information or Facts (% RNDs)	Cooking Methods/Recipes (% RNDs)	Counselling, Planning, and/or Professional Activity Promotion (% RNDs)
Dunne et al. (2019) [11]	WhatsApp (69%) Facebook (46%) Twitter (79%) Instagram (21%) LinkedIn (10%)	WhatsApp (59%) Facebook (41%) Twitter (44%) Instagram (44%) LinkedIn (3%)	WhatsApp (44%) Facebook (10%) Twitter (5%) Instagram (5%) LinkedIn (3%)
Zielińska-Tomczak et al. (2021) [8]	Instagram (100%) ^a^	Not mentioned	Not mentioned
Sbardelotto et al. (2022) [5]	Social network not specified (18–57.6%) ^b^	Not mentioned	Social network not specified (25–39.6%)
Squires et al. (2023) [9]	Instagram (49%)	Instagram (21%)	Instagram (19%)
Bookari et al. (2023) [6]	Social network not specified BC (35.7%) AC (44.1%)	Social network not specified BC (17.5%) AC (21.6%)	Social network not specified BC (51.1%) AC (63%)
Liikkanen et al. (2024) [13]	Facebook (20%) Twitter (46%) Instagram (27%) LinkedIn (24%)	Facebook (24%) Twitter (36%) Instagram (46%) LinkedIn (21%)	YouTube (17%) Instagram (4.5%) Facebook/Twitter/LinkedIn/WhatsApp (0%)
Visser et al. (2024) [14]	Social network not specified 62.6%	Social network not specified 29.3%	Facebook (60.6%) Twitter (9.1%) Instagram (53.5%) LinkedIn (22.2%)

(BC = Before COVID-19; AC = After COVID-19); ^a^ only includes nutrition information; no mention of other categories. ^b^ Participants reported engaging in these activities (‘always’ to ‘almost always’) within a range of responses without specifying the social network used.

**Table 6:** Enablers, Barriers and Perceived Benefits of Social Media Use reported by RNDs.

Authors	Motivating Factors/Enablers	Challenges/Barriers	Importance of Use/Opinions/Benefits
Dunne et al. (2019) [11]	Better communication with customers Improved client mobile learning	Insufficient training in digital interventions	–
Sbardelotto et al. (2022) [5]	–	–	Social media is a good tool to promote services
Saboia et al. (2021) [10]	–	–	Networking with peer professionals Dissemination of knowledge on healthy eating Sharing evidence-based nutrition content Engagement with individuals interested in nutrition
Liikkanen et al. (2024) [13]	Inclusion of social media in the workplace Measurable advantages of social media use Improved social media skills- Appropriate communication environment Effective self-created content	–	–
Visser et al. (2024) [14]	Better reach through digital platforms User-friendly and accessible Use of platforms preferred by younger audiences	Lack of time Unfamiliarity with some platforms Limited active participation	–
Bookari et al. (2023) [6]	–	Time constraints Communication challenges Anthropometric assessments compromised Connectivity issues Limited social media experience Restricted access to paid apps Lack of face-to-face interaction	Quick and efficient information exchange Broad reach in a short time Networking with colleagues Flexible and location Independent Cost-effective

## Data Availability

The original contributions presented in this study are included in the article. Further inquiries can be directed to the corresponding author.

## Abbreviations

The following abbreviations are used in this manuscript:

RNDs	Registered Nutritionists and Dietitians
MMAT	Mixed Methods Appraisal Tool
HPCSA	Health Professions Council of South Africa
